# Validated Reversed-Phase Ion-Interaction High-Performance Liquid Chromatography for Quantitation of Nitrate Content of *Clausena anisata* (Willd.) Hook. f. ex Benth. Leaves

**DOI:** 10.1155/2020/6424682

**Published:** 2020-03-14

**Authors:** Chaowalit Monton, Jirapornchai Suksaeree, Chitradee Luprasong

**Affiliations:** ^1^Drug and Herbal Product Research and Development Center, College of Pharmacy, Rangsit University, Pathum Thani 12000, Thailand; ^2^Department of Pharmaceutical Chemistry, College of Pharmacy, Rangsit University, Pathum Thani 12000, Thailand; ^3^Sun Herb Thai Chinese Manufacturing, College of Pharmacy, Rangsit University, Pathum Thani 12000, Thailand

## Abstract

This work sought to validate the reversed-phase ion-interaction high-performance liquid chromatography for quantifying the nitrate content in the extract and raw material of *Clausena anisata* (Willd.) Hook. f. ex Benth. leaves. Three extraction methods (i.e., decoction, infusion, and ultrasound-assisted extraction) were investigated and compared. Furthermore, the effect of the solid-to-solvent ratio and defatting was also evaluated. The validation result showed that the high-performance liquid chromatographic method had a linear response (*R*^2^ = 0.9999) in the range of 1–50 *μ*g/mL. The limit of detection and limit of quantitation were 0.25 *μ*g/mL and 0.75 *μ*g/mL, respectively. In addition, the method was specific, precise, and accurate. So the validated method was suitable for determination of the nitrate content in *C. anisata* leaves. Infusion of a nondefatted sample using a solid-to-solvent ratio of 1 : 10 gave the highest nitrate content in the raw material, 0.251 ± 0.003%. In case of a defatted sample, decoction provided the highest nitrate content, 0.309 ± 0.001%. Increasing the solid-to-solvent ratio and defatting had a huge effect on the nitrate content of *C. anisata* leaves extracted from decoction. To the best of our knowledge, this is the first report that used the reversed-phase ion-interaction high-performance liquid chromatography for quantifying the nitrate content in *C. anisata* leaves. Furthermore, the authors suggested that nitrate could be used as a standard marker for quality control of *C. anisata* leaves' extract and raw material.

## 1. Introduction

Extraction is an important step in the analysis of plant composition and investigation of its biological and pharmacological activity. The appropriate extraction technique provides the high content of desired plant bioactive compounds as well as prevents the decomposition of labile compounds [1]. According to decoction and infusion, there are classical extraction techniques which usually use traditional medicines due to the fact that they are simple and easy methods. Decoction and infusion are suitable for extraction of heat-stable compounds [2]. Ultrasound-assisted extraction (UAE) or sonication is a modern extraction technique. It was developed for the reduction of time, energy, and solvent consumption [3, 4]. Extraction technique plays an important role in outcomes such as the yield and content of active compounds. So a selection of extraction procedure is an important step to be considered.

*Clausena anisata* (Willd.) Hook. f. ex Benth. is a plant in family Rutaceae. *C. anisata* leaves possess several biological activities including analgesic [5], antidiabetic [6, 7], anti-inflammatory [8, 9], antimalarial [5], antimicrobial [7, 10–13], antioxidant [7, 14], antipyretic [8], cytotoxic [10], and larvicidal [15–19]. *C. anisata* leaves are rich in volatile oil, so volatile oil composition is usually studied. (E)-Anethole is reported as a major constituent of volatile oil of *C. anisata* [20, 21]. However, some varieties contain methyl chavicol as a major composition [20, 22].

Smoking causes health problems around the world. In 2011, tobacco use caused approximately six million deaths annually. The current trend reveals that tobacco use will kill more than eight million deaths per year in 2030 [23]. Smoking causes numerous diseases, i.e., cancers, respiratory diseases, cardiovascular diseases, oral diseases, impaired vision, bone fractures, and diabetes [24]. During 1965–2014, smoking and exposure to secondhand smoke caused approximately 21 million premature deaths [25]. Many modern medicinal products are developed to support smoking cessation. However, some herbal plants are also used as an aid for smoking cessation. In Thailand, *C. anisata* leaves are used as a smoking cessation aid similar to a well-known smoking cessation aid plant: *Vernonia cinerea* (L.) Less. Formerly, *V. cinerea* was reported to contain nitrate salt that can induce tongue numbness and cause less favor of cigarette smell and taste [26, 27]. However, the nitrate content in *C. anisata* leaves has not been reported. We expected that *C. anisata* leaves may also contain nitrate similar to *V. cinerea*. Furthermore, the high content of nitrate was related to the quality of the plant used for smoking cessation. Thus, the nitrate content can be selected as a standard marker for quality control of *C. anisata* leaves for smoking cessation use. To the best of our knowledge, there is no report about the high-performance liquid chromatography (HPLC) method of quantitation of the nitrate content of *C. anisata* leaves. So, the aim of this work was quantitation of the nitrate content of *C. anisata* leaves using validated reversed-phase ion-interaction HPLC. The nitrate content of *C. anisata* leaves extracted by different techniques and solid-to-solvent ratio were compared. Furthermore, the nitrate content of nondefatted and defatted *C. anisata* leaves was also compared to determine the best extraction condition that provided the highest content of nitrate. The authors expected that nitrate could be used as a standard marker for quality control of *C. anisata* leaves' extract and raw material. Moreover, the suitable extraction method and extraction condition could be selected to extract the *C. anisata* leaves to improve further product development.

## 2. Materials and Methods

### 2.1. Materials

Sodium nitrate (purity 99%) and 85% orthophosphoric acid were purchased from Carlo Erba Reagents, France. Octylamine was purchased from Sigma-Aldrich, USA. Hexane (AR grade) was purchased from Duksan Pure Chemicals, Korea. Methanol (HPLC grade) was purchased from Honeywell-Burdick & Jackson, USA.

### 2.2. Plant Sample

*C. anisata* (Willd.) Hook. f. ex Benth. leaves were harvested from Sing Buri Province in November 2018. The plant sample was identified to ensure the right species by Chair Prof. Dr. Nijsiri Ruangrungsi, Department of Pharmacognosy, College of Pharmacy, Rangsit University. Voucher specimen no. CM-CA003-1-11-2018 was deposited at Drug and Herbal Product Research and Development Center, College of Pharmacy, Rangsit University.

### 2.3. Extraction Procedures

*C. anisata* leaves were sun-dried and ground using a grinder. Nondefatted and defatted *C. anisata* leaf powder was extracted using three different techniques (i.e., decoction, infusion, and UAE). Defatted samples were defatted by hexane: 100 g of *C. anisata* leaf powder was mixed with 500 mL hexane and sonicated for 30 min. It was defatted with new hexane three times. Then, the defatted *C. anisata* leaf powder was dried at ambient conditions for 2 h prior to extraction; the moisture content of defatted and nondefatted samples measured by a moisture analyzer was 11.27 ± 0.03% and 10.77 ± 0.17%, respectively. In case of decoction, boiling water was added to *C. anisata* leaf powder and was boiled for 15 min. In case of infusion, boiling water was added to *C. anisata* leaf powder and was allowed to stand at ambient temperature for 15 min. In case of UAE, water at ambient temperature was added to *C. anisata* leaf powder, which was sonicated in an ultrasonic bath (frequency 40 kHz, power 500 W, temperature ≤30°C) for 15 min. Each extraction technique was performed three times. The three filtrates were pooled and lyophilized. When a solid-to-solvent ratio of 1 : 10 was performed, 5 g of *C. anisata* leaf powder and 50 mL of the solvent were used. In contrast, 5 g of *C. anisata* leaf powder and 100 mL of the solvent were used for the solid-to-solvent ratio of 1 : 20. However, a solid-to-solvent ratio of 1 : 10 was applied for the nondefatted sample, while a solid-to-solvent ratio of 1 : 20 was applied for both nondefatted and defatted samples. The resulting extracts were kept in a desiccator until use.

### 2.4. Analysis of Nitrate Content and Method Validation

The HPLC used for the analysis of the nitrate content was adapted from Cheng and Tsang [28] and Chou et al. [29]. They used this method to determine the nitrate content in canned vegetable juices and vegetables. The HPLC condition used for the analysis of the nitrate content in *C. anisata* was performed according to our previous works [30, 31]. Reversed-phase ion-interaction HPLC was applied in this work. The isocratic system was performed on an ACE Generix column (150 × 4.6 mm, i.d., 5 *μ*m) with temperature controlled at 25°C. 0.01 M octylammonium orthophosphate (pH 7.0) was used as the mobile phase. It was prepared by dissolving 1.3 g octylamine in 30% v/v methanol, the pH was adjusted to 7.0 by 10% orthophosphoric acid, and the volume was adjusted to 1,000 mL with 30% v/v methanol. The flow rate of the mobile phase was 0.8 mL/min. The injection volume was 10 *μ*L. The detection wavelength was 213 nm. The sample was analyzed after the injections of the standard solution provided the reproducible retention time as well as the peak area. The content of nitrate in the extract and in *C. anisata* leaf powder (it was called the *C. anisata* raw material) was calculated from the calibration curve of standard nitrate.

Method validation was investigated with regard to several variables including linearity, range, specificity, limit of detection (LOD) and limit of quantitation (LOQ), precision, and accuracy. According to linearity, a stock solution of nitrate in a concentration of 1 mg/mL was prepared using water as a solvent. It was diluted into a concentration range of 50, 25, 10, 5, and 1 *μ*g/mL. They were filtered and analyzed by HPLC. Specificity was determined by comparison of UV spectrums of standard nitrate and nitrate in the *C. anisata* extract at upslope, top, and downslope of the peak. The HPLC method was specific when all UV spectrums were similar. LOD and LOQ of the analysis were calculated based on standard deviation (SD) of y-intercepts of regression lines and slope of the calibration curve as described in the previous work [31]. Precision of the analysis was reported as percent relative SD (percent RSD) of intraday and interday analysis. Three concentrations of nitrate solution were prepared, 5, 10, and 25 *μ*g/mL. They were analyzed by HPLC: the analysis in the same day was reported as intraday precision and analysis in the three consecutive days was reported as interday precision. The percent RSD of intraday and interday precision should be less than 2% and 5%, respectively. Finally, accuracy was determined using the standard addition technique. Nitrate solution in the concentration of 5, 10, and 25 *μ*g/mL was added into the *C. anisata* extract with a known concentration of nitrate (5 *μ*g/mL). In this case, the final concentrations of nitrate in the mixtures were 10, 15, and 30 *μ*g/mL. They were analyzed by HPLC, and percent recovery of each concentration level was calculated.

## 3. Results

**Figure 1:**
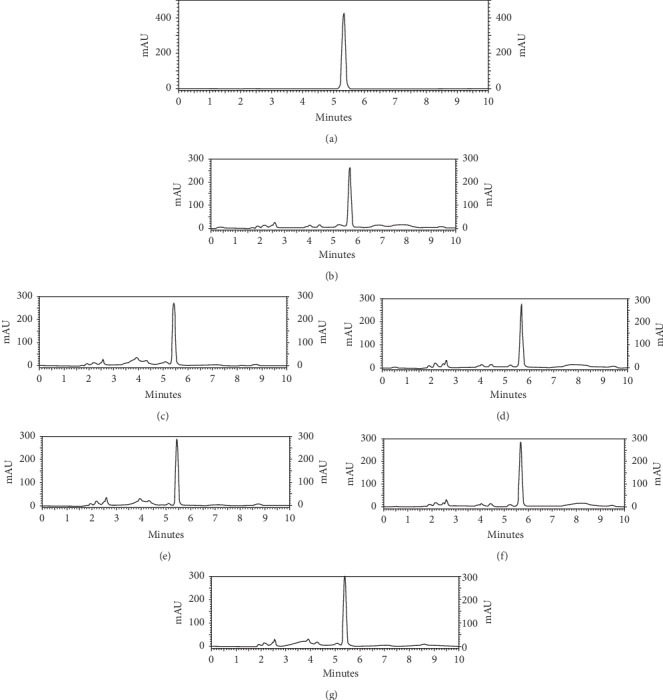
HPLC chromatograms of (a) standard nitrate (25 *μ*g/mL): nondefatted *C. anisata* extract (2.5 mg/mL) obtained from (b) decoction, (d) infusion, and (f) UAE and defatted *C. anisata* extract (2.5 *m*g/mL) obtained from (c) decoction, (e) infusion, and (g) UAE.

**Figure 2:**
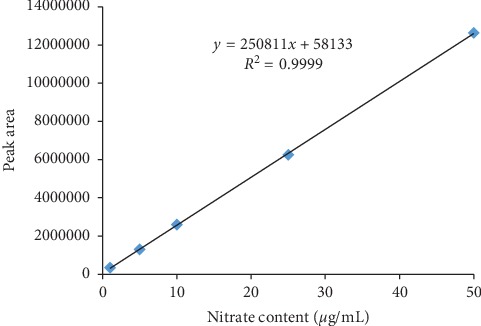
Calibration curve of nitrate for analysis of the nitrate content in *C. anisata* leaves.

**Figure 3:**
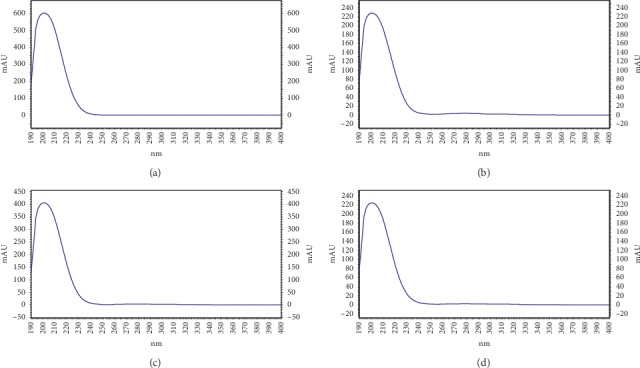
UV spectrums of (a) standard nitrate and nitrate in the *C. anisata* extract at (b) upslope, (c) top, and (d) downslope of the peak.

The HPLC method used in this work was previously used to determine the nitrate content in canned vegetable juices [28], vegetables [29], and sodium nitrate mouthwash [30, 31]. According to this work, the HPLC method was also applied to determine the nitrate content in *C. anisata* leaves.  Figure 1 shows the HPLC chromatogram of standard nitrate and nondefatted and defatted *C. anisata* leaf extracts obtained from different extraction techniques. The similar pattern of HPLC chromatograms was found for both nondefatted and defatted samples.  Figure 2 shows a calibration curve of nitrate in the test range of 1–50 *μ*g/mL. The linear equation and *R*^2^ are also shown. According to specificity, UV spectrums of standard nitrate and nitrate in the *C. anisata* extract at upslope, top, and downslope of the peak are shown in Figure 3. The UV spectrums of standard nitrate and nitrate in the *C. anisata* extract were similar, so this result indicated that the HPLC method was specific. LOD and LOQ calculated based on SD of y-intercepts of regression lines and slope of the calibration curve were 0.25 *μ*g/mL and 0.75 *μ*g/mL, respectively.  Table 1 shows the precision and accuracy of the analysis. Percent RSD of intraday and interday precision was less than 2%. This result indicated that the HPLC method was precise. In addition, the accuracy of the analysis represented as percent recovery showed that it was close to 100%, indicating that the HPLC method for analysis of nitrate was accurate.

**Figure 4:**
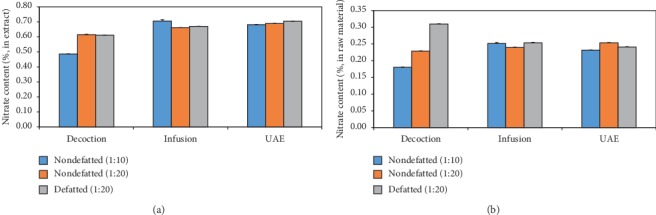
Nitrate content of the *C. anisata* leaf (a) extract and (b) raw material extracted from different techniques.

Extraction yields of nondefatted and defatted *C. anisata* leaves were in the range of 30–50%. Extraction yields of nondefatted *C. anisata* leaves obtained from decoction, infusion, and UAE using a solid-to-solvent ratio of 1 : 10 were 37.1%, 35.6%, and 33.9%, respectively. Using a solid-to-solvent ratio of 1 : 20 gave the extraction yield of 37.2%, 36.2%, and 36.8%, respectively. According to defatted groups, their extraction yield was 50.6%, 37.8%, and 34.2%, respectively.  Figure 4 shows the effect of extraction methods, solid-to-solvent ratio, and defatting on the nitrate content of *C. anisata* leaf extract and raw material. Infusion of nondefatted *C. anisata* leaves using a solid-to-solvent ratio of 1 : 10 provided the extract with the highest content of nitrate compared to UAE and decoction (Figure 4(a), blue bars). Among the three extraction methods, decoction extracted the lowest amount of nitrate. This order was similar for the nitrate content in the raw material (Figure 4(b), blue bars). The order of the nitrate content of nondefatted *C. anisata* leaves extracted using the solid-to-solvent ratio of 1 : 20 was similar in the extract and in the raw material. UAE provided a slightly higher nitrate content compared to infusion and decoction, respectively (Figure 4, orange bars). According to defatted samples, UAE provided the extract with the highest nitrate content compared to infusion and decoction, respectively (Figure 4(a), gray bars). However, the nitrate content in the raw material showed the highest value in the decoction group compared to infusion and UAE groups, respectively (Figure 4(b), gray bars). The conversion from the nitrate content in the extract into the nitrate content in the raw material was dependent on the extraction yield. In this case, the highest extraction yields were found in the defatted sample extracted by decoction, and so these sample also provided the highest nitrate content in the raw material. The solid-to-solvent ratio affected the nitrate content in the *C. anisata* leaf extract and raw material. In Figure 4, the solid-to-solvent ratios of 1 : 10 and 1 : 20 are investigated; the blue bar and orange bar for each extraction method are compared. Increasing the solvent ratio of decoction and UAE seems to increase the nitrate content of the *C. anisata* leaf extract and raw material, while it slightly decreases the nitrate content obtained from the infusion. The last evaluation factor was defatting of *C. anisata* leaves before extraction. So, the orange bar and gray bar are compared (Figure 4). *C. anisata* contained oil droplets in its leaves that might decrease the solubility of nitrate, a hydrophilic substance. So defatting perhaps increased the extraction efficiency of nitrate from *C. anisata* leaves. Figure 4(a) shows that defatting could slightly increase the nitrate content in the extract obtained from infusion and UAE, but it was comparable to that obtained by decoction. According to the nitrate content in the raw material, defatting had a large positive effect on the nitrate content obtained from decoction. Defatting slightly affected the infusion, but it did not affect the UAE (Figure 4(b)).

## 4. Discussion

The authors succeeded in the validation of reversed-phase ion-interaction HPLC to quantitate the nitrate content of *C. anisata* leaves. This is the first work to report the determination method of nitrate in *C. anisata* leaves by using reversed-phase ion-interaction HPLC. This method showed a linear response and specific, precise, and accurate style. Moreover, this method was easy and rapid [28, 29, 32]. The advantages of this method were the high stability and efficiency of the columns and the lack of the organic stationary phase to be regenerated [32]. So, it could be applied to quantitation of the nitrate content in other plants or products.

Besides *C. anisata*, *V. cinerea* and *M. siamensis* also contained nitrate. *V. cinerea* stem extracts and leaf extracts contained the nitrate content of 21% and 19%, respectively [33], which were higher than those of *C. anisata* investigated in this work. Wongsasjanan et al. [34] evaluated the effect of the harvesting period on the nitrate content of *M. siamensis*leaves. They found that the nitrate content in 45-day *M. siamensis* leaves was greatest compared to that in 60-day and 30-day leaves, 7.50 mg/L, 7.15 mg/L, and 6.87 mg/L, respectively. We calculated from the data shown in this paper that the extract of 30-day, 45-day, and 60-day groups contained nitrate of 0.344%, 0.375%, and 0.358%, respectively. The same colleagues also investigated the effect of fertilizers on the nitrate content of *M. siamensis* leaves. They reported that the leaf extract of the 60-day *M. siamensis* group that received the chemical fertilizer contained the nitrate content of 7.32 mg/L. The extract of *M. siamensis* that received the natural fertilizer and no fertilizer contained nitrate of 6.22 mg/L and 6.11 mg/L [35], which were equivalent to 0.366%, 0.311%, and 0.301%, respectively. The nitrate content in the *M. siamensis* leaf extract was lower than that in the *C. anisata* leaf extract reported in our work.

The extraction method affected the extraction of plant bioactive compounds. Different extraction techniques provided the different content of individual compounds as well as their biological or pharmacological activities. Kaneria et al. [36] showed that extraction techniques and solvent types affected the content of antioxidant compounds and antioxidant activity of pomegranate leaves and stems. Successive cold percolation, individual cold percolation, and decoction were compared. Successive cold percolation with acetone could extract the highest content of total phenolic compounds. In contrast, decoction and individual cold percolation using water could extract comparable contents of total phenolic compounds. Furthermore, the content of total phenolic compounds was associated with the antioxidant activity of the pomegranate leaf and stem extract. The effect of the extraction method and solvent type on the phenolic compound and antioxidant activity was also investigated in *Xanthium strumarium* leaves. Three extraction methods were compared (i.e., static maceration, dynamic maceration, and Soxhlet extraction). Results showed that the extraction method affected the extraction yield, total phenolic content, antioxidant activity, and content of individual phenolic compounds (i.e., chlorogenic acid, ferulic acid, and *trans*-cinnamic acid) [37]. In addition, the extraction technique and solvent type also affected the extraction yield, total phenolic content, withanolide A content, 12-deoxy withastramonolide content, and antioxidant activity of roots of *Withania somnifera* [38]. Volatile oil of *Tetraclinis articulata* leaves was also affected by the extraction method. Volatile oil of *T. articulata*leaves from microwave-assisted hydrodistillation contained more oxygenated compounds, while conventional hydrodistillation gave more hydrocarbon compounds. Furthermore, volatile oil obtained from microwave-assisted hydrodistillation showed superior antioxidant activity and anti-inflammatory activity compared to that from conventional hydrodistillation [39].

Typically, the use of more solvent could increase the extraction yield and content of plant bioactive compounds. However, the use of more solvent could increase the cost and time of solvent elimination from the extract. The effect of the solid-to-solvent ratio (1 : 5 to 1 : 20) was investigated on the total phenolic content and total flavonoid content of *Centella asiatica*. The optimal solid-to-solvent ratio was 1 : 15. Further increases in the solvent ratio did not significantly increase the total phenolic content or total flavonoid content [40]. The solid-to-solvent ratio of 1 : 20 could maximize the total phenolic content and total flavonoid content of *Phyllanthus niruri* [41]. Radojković et al. [42] optimized the solid-to-solvent ratio for the extraction of black mulberry leaves. The solid-to-solvent ratio was varied from 1 : 10 to 1 : 30. They found that the solid-to-solvent ratio of 1 : 20 could extract the maximum total phenolic compounds similar to the total flavonoid content. Zubairi et al. [43] suggested that increasing the solvent ratio had a positive effect on the rotenone content of *Derris elliptica* roots. The result showed that increasing the solvent ratio from 1 : 3.3 to 1 : 10 yielded more rotenone content. The result was similar to a report of Predescu et al. [44]; increasing the solvent ratio from 1 : 5 to 1 : 10 gave more total phenolic content and total flavonoid content of dog-rose fruits, sea buckthorn fruits, and hawthorn fruits. Moreover, Said et al. [45] revealed that the solid-to-solvent ratio of 1 : 10 was more suitable for extraction of vitamin C from banana peel than the solid-to-solvent ratio of 1 : 4.5 and 1.5. These results were similar to our work that increasing the solvent ratio could increase the nitrate content in decoction and UAE groups.

Defatting could be useful in the extraction of the hydrophilic compound from plants by decreasing hydrophobicity of the plant matrix. However, defatting using an organic solvent such as hexane might destroy or could extract some bioactive compounds of the plant. Saikusa et al. [46] suggested that defatting could reduce rancidity of rice germ. Defatting with hexane did not affect *γ*-aminobutyric acid accumulation in rice germ, but that with ethanol decreased the *γ*-aminobutyric acid content. Buitimea-Cantúa et al. [47] reported that defatting of sorghum bran fraction with hexane could decrease phenolic compounds compared to the nondefatted group. They described that defatting could solubilize nonpolar phenolic compounds of sorghum bran fraction. Our work found that defatting had less effect on infusion and UAE but showed a great effect on decoction. This phenomenon could be described by the fact that defatting could eliminate the oil component in *C. anisata* leaf powder, so nitrate could be easily extracted from the low hydrophobic plant matrix.

## 5. Conclusions

The reversed-phase ion-interaction HPLC system was validated. It showed a linear response and was specific, precise, and accurate. So it was used to analyze the nitrate content in the extract and raw material of *C. anisata* leaves. Infusion of the nondefatted sample using a solid-to-solvent ratio of 1 : 10 provided the highest nitrate content in the extract and raw material. Among the three extraction methods of the defatted sample, decoction was the best extraction method that could extract the highest nitrate content. Increasing the solvent ratio could increase the nitrate content in decoction and UAE. Defatting had less effect on infusion and UAE but showed a great effect on decoction. In summary, the reversed-phase ion-interaction HPLC could be applied to the analysis of the nitrate content. In addition, this work supported that nitrate could be used as a standard marker for quality control of extracts, raw materials, or smoking cessation products of *C. anisata*.

## Figures and Tables

**Table 1:** Precision and accuracy of nitrate analysis.

Concentration (*μ*g/mL)	Precision (percent RSD)^*∗*^				Spike concentration (*μ*g/mL)	Accuracy
	Intraday			Interday		Recovery (%)
	Day 1	Day 2	Day 3			
5	1.50	0.11	0.10	1.88	5	98.11 ± 0.02
10	0.08	0.71	0.04	1.11	10	96.32 ± 0.00
25	0.07	0.04	0.04	1.32	25	98.27 ± 0.00

^*∗*^Percent RSD = SD × 100/mean.

## Data Availability

The data used to support the findings of this study are available from the corresponding author upon request.

## References

[1] Sasidharan S., Chen Y., Saravanan D., Sundram K., Latha L. (2010). Extraction, isolation and characterization of bioactive compounds from plants’ extracts. *African Journal of Traditional, Complementary and Alternative Medicines*.

[2] Azwanida N. N. (2015). A review on the extraction methods use in medicinal plants, principle, strength and limitation. *Medicinal and Aromatic Plants Research Journals*.

[3] Azmir J., Zaidul I. S. M., Rahman M. M. (2013). Techniques for extraction of bioactive compounds from plant materials: a review. *Journal of Food Engineering*.

[4] Zhang Q.-W., Lin L.-G., Ye W.-C. (2018). Techniques for extraction and isolation of natural products: a comprehensive review. *Chinese Medicine*.

[5] Okokon J. E., Etebong E. O., Udobang J. A., Essien G. E. (2012). Antiplasmodial and analgesic activities of *Clausena anisata*. *Asian Pacific Journal of Tropical Medicine*.

[6] Mogale M. A., Mkhombo H. M., Lebelo S. L. (2012). The effects of *Clausena anisata* (Wild) Hook leaf extracts on selected diabetic related metabolizing enzymes. *Journal of Medicinal Plants Research*.

[7] Arsia Tarnam Y., Nargis Begum T., Deepa N. S. (2014). Study of *in vitro* antioxidant, antidiabetic and antibacterial activity of *Clausena anisata* (Willd). Hook (Rutaceae) leaf extracts. *Journal of Pharmacology and Pharmacotherapeutics*.

[8] Okokon J. E., Udoh A. E., Andrew U. E. (2012). Antiinflammatory and antipyretic activities of *Clausena anisata*. *Molecular and Clinical Pharmacology*.

[9] Jeon C.-M., Shin I.-S., Shin N.-R. (2016). *Clausena anisata*-mediated protection against lipopolysaccharide-induced acute lung injury in mice. *International Journal of Molecular Medicine*.

[10] Makirita W., Chauka L., Chacha M. (2016). Antimicrobial and cytotoxicity activity of *Clausena anisata, Acokanthera shemperii* and *Olea europaea* growing in Tanzania. *European Journal of Medicinal Plants*.

[11] Agyepong N., Agyare C., Adarkwa-Yiadom M., Gbedema S. Y. (2014). Phytochemical investigation and anti-microbial activity of *Clausena anisata* (Willd.) Hook.. *African Journal of Traditional, Complementary and Alternative Medicines*.

[12] Lawal I. O., Galadima M., Ogunbamowo P. O. (2018). Isolation of bioactive compounds of *Clausena anisata* (Willd.) Hook. growing in South Africa by liquid chromatography–mass spectroscopy profiling, and their antibacterial activities. *Journal of Medicinal Plants for Economic Development*.

[13] Senthilkumar A., Venkatesalu V. (2009). Phytochemical analysis and antibacterial activity of the essential oil of *Clausena anisata* (Willd.) Hook. f. ex Benth.. *International Journal of Integrative Biology*.

[14] Lawal I. O., Grierson D. S., Afolayan A. J. (2015). Phytochemical and antioxidant investigations of a *Clausena anisata* hook, a South African medicinal plant. *African Journal of Traditional, Complementary and Alternative Medicines*.

[15] Mukandiwa L., Ahmed A., Eloff J. N. (2013). Isolation of seselin from *Clausena anisata* (Rutaceae) leaves and its effects on the feeding and development of *Lucilia cuprina* larvae may explain its use in ethnoveterinary medicine. *Journal of Ethnopharmacology*.

[16] Mukandiwa L., Eloff J. N., Sibanda D. R. (2016). An acetone extract of *Clausena anisata* may be a potential control agent for flies encountered in cutaneous myiasis. *Onderstepoort Journal of Veterinary Research*.

[17] Pavela R., Maggi F., Lupidi G. (2018). *Clausena anisata* and *Dysphania ambrosioides* essential oils: from ethno-medicine to modern uses as effective insecticides. *Environmental Science and Pollution Research*.

[18] Aurelie F. D. G., Pierre B. N. M., Ascension N. M. (2018). Chemical composition and biocide properties of *Clausena anisata* (Rutaceae) essential oil against developmental stages of the malaria vector *Anopheles coluzzii*. *American Journal of Essential Oils and Natural Products*.

[19] Govindarajan M. (2010). Chemical composition and larvicidal activity of leaf essential oil from *Clausena anisata* (Willd.) Hook. f. ex Benth (Rutaceae) against three mosquito species. *Asian Pacific Journal of Tropical Medicine*.

[20] Addae-Mensah I., Asomaning W. A., Oteng-Yeboah A. (1996). (E)-anethole as a major essential oil constituent of *Clausena anisata*. *Journal of Essential Oil Research*.

[21] Usman L. A., Hamid A. A., Olawore N. O. (2010). Chemical composition of leaf essential oil of *Clausena anisata* growing in North-Central Nigeria. *Journal of Applied Sciences Research*.

[22] Ekundayo O., Oguntimein B. O., Hammerschmidt F. J. (1986). Constituents of the essential oil of *Clausena anisata* leaves. *Planta Medica*.

[23] Centers for Disease Control and Prevention (2018). *Smoking & Tobacco Use*.

[24] WorkSHIFTS (2011). *Diseases and Conditions Related to Smoking*.

[25] US Department of Health and Human Services, *The Health Consequences of Smoking—50 Years of Progress: A Report of the Surgeon General*, Centers for Disease Control and Prevention, Atlanta, GA, USA, 2014, https://www.surgeongeneral.gov/library/reports/50-years-of-progress/exec-summary.pdf

[26] Chaikoolvatana A., Ayuthaya N. D., Suthipinittharm P. (2017). Development and evaluation of the effectiveness of *Vernonia cinerea* (VC) cookies for smoking cessation. *Journal of Health Research*.

[27] Chaikoolvatana A., Thanawirun J., Chaikoolvatana C. (2018). Use of *Vernonia cinerea* jelly candies for smoking cessation, Ubon Ratchathani region, Thailand. *EnvironmentAsia*.

[28] Cheng C. F., Tsang C. W. (1998). Simultaneous determination of nitrite, nitrate and ascorbic acid in canned vegetable juices by reverse-phase ion-interaction HPLC. *Food Additives & Contaminants*.

[29] Chou S.-S., Chung J.-C., Hwang D.-F. (2003). A high performance liquid chromatography method for determining nitrate and nitrite levels in vegetables. *Journal of Food and Drug Analysis*.

[30] Monton C., Charoenchai L., Suksaeree J. (2017). Forced degradation study of sodium nitrate solution and its formulation. *Thai Journal of Pharmaceutical Sciences*.

[31] Monton C., Boonkrungthong B., Settharaksa S. (2016). Method validation and quantitation of sodium nitrate in 0.5% sodium nitrate mouthwash for smoking cessation. *Bulletin of Health Science and Technology*.

[32] Fallon A., Booth R. F. G., Bell L. D., Fallon A., Booth R. F. G., Bell L. D. (1987). High performance reverse phase ion-pair chromatography. *Laboratory Techniques in Biochemistry and Molecular Biology*.

[33] Ketsuwan N., Leelarungrayub J., Kothan S. (2017). Antioxidant compounds and activities of the stem, flower, and leaf extracts of the anti-smoking Thai medicinal plant: *Vernonia cinerea* Less. *Drug Design, Development and Therapy*.

[34] Wongsasjanan S., Mungngam J., Kaewsri P. (2016). The study of harvesting period to yields and nitrate production of *Murraya siamensis*’s leaves. *Songklanakarin Journal of Science and Technology*.

[35] Chindachia R., Mungngam J., Wongsasjanan S. (2016). The study of fertilizer applications to yields and concentration of nitrate of *Murraya siamensis*’s leaves at age 30 and 60 days of harvest. *Songklanakarin Journal of Science and Technology*.

[36] Kaneria M. J., Bapodara M. B., Chanda S. V. (2012). Effect of extraction techniques and solvents on antioxidant activity of pomegranate (*Punica granatum* L.) leaf and stem. *Food Analytical Methods*.

[37] Scherer R., Godoy H. (2014). Effects of extraction methods of phenolic compounds from *Xanthium strumarium* L. and their antioxidant activity. *Revista Brasileira de Plantas Medicinais*.

[38] Dhanani T., Shah S., Gajbhiye N. A. (2017). Effect of extraction methods on yield, phytochemical constituents and antioxidant activity of *Withania somnifera*. *Arabian Journal of Chemistry*.

[39] Djouahri A., Boudarene L., Meklati B. Y. (2013). Effect of extraction method on chemical composition, antioxidant and anti-inflammatory activities of essential oil from the leaves of Algerian *Tetraclinis articulata* (Vahl) Masters. *Industrial Crops and Products*.

[40] Tan P. W., Tan C. P., Ho C. W. (2011). Antioxidant properties: effects of solid-to-solvent ratio on antioxidant compounds and capacities of Pegaga (*Centella asiatica*). *International Food Research Journal*.

[41] Wong B. Y., Tan C. P., Ho C. W. (2013). Effect of solid-to-solvent ratio on phenolic content and antioxidant capacities of “Dukung Anak” (*Phyllanthus niruri*). *International Food Research Journal*.

[42] Radojković M., Zeković Z., Jokić S. (2012). Optimization of solid-liquid extraction of antioxidants from black mulberry leaves by response surface methodology. *Food Technology and Biotechnology*.

[43] Zubairi S. I., Sarmidi M. R., Aziz R. A. (2014). The effects of raw material particles size, types of solvents and solvent-to-solid ratio on the yield of rotenone extracted from *Derris elliptica* roots. *Sains Malaysiana*.

[44] Predescu N. C., Papuc C., Nicorescu V. (2016). The influence of solid-to-solvent ratio and extraction method on total phenolic content, flavonoid content and antioxidant properties of some ethanolic plant extracts. *Revista de Chimie*.

[45] Said K. A. M., Yakub I., Alipah N. A. M. (2016). Effects of solvent/solid ratio and temperature on the kinetics of vitamin C extraction from *Musa acuminata*. *Applied Mechanics and Materials*.

[46] Saikusa T., Okada T., Murai H. (2001). The effect of defatting with organic solvent on accumulation of 4-aminobutyric acid (GABA) in the rice germ. *Nippon Shokuhin Kagaku Kogaku Kaishi*.

[47] Buitimea-Cantúa N. E., Torres-Chávez P. I., Ledesma-Osuna A. I. (2013). Effect of defatting and decortication on distribution of fatty acids, phenolic and antioxidant compounds in sorghum (*Sorghum bicolor*) bran fractions. *International Journal of Food Science & Technology*.

